# No evidence for local adaptation to salt stress in the existing populations of invasive *Solidago canadensis* in China

**DOI:** 10.1371/journal.pone.0175252

**Published:** 2017-04-06

**Authors:** Junmin Li, Haiyan Liu, Ming Yan, Leshan Du

**Affiliations:** 1 Zhejiang Provincial Key Laboratory of Evolutionary Ecology and Conservation, Taizhou University, Taizhou, Zhejiang, China; 2 School of Life Science, Shanxi Normal University, Linfen, Shanxi, China; 3 Chinese Research Academy of Environmental Sciences, Beijing, China; Shandong University, CHINA

## Abstract

Local adaptation is an important mechanism underlying the adaptation of plants to environmental heterogeneity, and the toxicity of salt results in strong selection pressure on salt tolerance in plants and different ecotypes. *Solidago canadensis*, which is invasive in China, has spread widely and has recently colonized alkali sandy loams with a significant salt content. A common greenhouse experiment was conducted to test the role of local adaptation in the successful invasion of *S*. *canadensis* into salty habitats. Salt treatment significantly decreased the growth of *S*. *canadensis*, including rates of increase in the number of leaves and plant height; the root, shoot, and total biomass. Furthermore, salt stress significantly reduced the net photosynthetic rate, stomatal conductance, transpiration rate and relative chlorophyll content but significantly increased peroxidase activity and the proline content of *S*. *canadensis* and the root/shoot ratio. Two-way analysis of variance showed that salt treatment had a significant effect on the physiological traits of *S*. *canadensis*, except for the intercellular CO_2_ concentration, whereas the population and the salt × population interaction had no significant effect on any physiological traits. Most of the variation in plasticity existed within and not among populations, excep for the root/shoot ratio. *S*. *canadensis* populations from soil with moderate/high salt levels grew similarly to *S*. *canadensis* populations from soils with low salt levels. No significant correlation between salt tolerance indices and soil salinity levels was observed. The plasticity of the proline content, intercellular CO_2_ concentration and chlorophyll content had significant correlations with the salt tolerance index. These findings indicate a lack of evidence for local adaption in the existing populations of invasive *S*. *canadensis* in China; instead, plasticity might be more important than local adaptation in influencing the physiological traits and salt tolerance ability across the *S*. *canadensis* distribution.

## Introduction

Invasive species are considered the second greatest threat to native biodiversity following habitat destruction and are a hotspot in the study of ecology and the environment [1]. When alien plants are introduced into a novel region, they confront novel abiotic and biotic factors that may determine invasion success [2, 3]. It has been well documented that invasive plants have stronger adaptation ability to heterogeneous environments than native species [4, 5], yet there is also evidence showing that invasive and native plants have equal ability to adapt to heterogeneous environments [6]. Nontheless, it remains unclear why and how invasive plants adapt to novel heterogeneous abiotic and biotic factors [7].

Because plants are sessile during a significant fraction of their life cycle and are subject to strong selection for optimal performance under local environmental conditions [8], local adaptation contributes to the successful occupation of different habitats and wide distribution [7, 9, 10]. Geographic variation can lead to the evolution of different adaptations to a local environment and thus generate ecotypic differentiation in important functional traits [11, 12]. Furthermore, local adaptation has been well verified as a major mechanism enhancing invasiveness [13], which helps invasive plants successfully adapt to novel heterogeneous environments [7, 9]. Alternatively, phenotypic plasticity, the ability of a single genotype to produce different phenotypes in response to environmental variation [14–16], also contributes to the success of an invasive plant species by allowing increased fitness across a range of habitats [3, 17–22] and plays an important role in successful establishment under novel conditions [19, 23]. A central issue in invasion ecology is determining the importance of phenotypic plasticity and local adaptation in the adaption of invasive plant to novel heterogeneous environments across its distribution. However, contradictory conclusions have been drawn, indicating that the importance of these factors might depend on the plant species (for phenotypic plasticity rather than local adaptation, see reference [3, 24]; for both phenotypic plasticity and local adaptation, see reference [13, 25]. Thus, more attention should be paid to invasive plants to explore the mechanisms underlying invasion success [24].

Soil salinity is a determining factor in species colonization because salt can be toxic [26]. It is well documented that salinity can inhibit the growth of plants through water deficit, ion toxicity, ion imbalance or a combination of these factors, and plants respond to salinity stress at physiological, biochemical, molecular and morphological levels [27, 28]. However, the effect of salinity on plant growth is dependent on the salt concentration. A high saline environment may reduce parameters, such as the photosynthetic rate, plant height, the number of leaves, root length and increase the root/shoot ratio [27], whereas low salinity may stimulate plant growth [28]. In addition, the effect of soil salinity on plant species varies among taxa [29], including invasive plants [26]. Both phenotypic plasticity and genetic adaptation may contribute to the salt tolerance of plants [7, 8], and soil salinity plays an important role in favoring or limiting the spread of alien species, especially in the colonization of saline environments [30]. For example, salinity was found to enhance the replacement of native *Spartina alterniflora* by invasive *Phragmites australis* [30] but to limit the replacement of native *Salicornia subterminalis* by invasive *Polypogon monspeliensis* [31]. Understanding the response of invasive plants to soil salinity when there is potential for colonizing saline environments is key to providing basic references for the management and control of invasive plants [30].

*Solidago canadensis* L. (Asteraceae), a long-lived rhizomatous perennial forb of North American origin, is one of the most widespread invasive alien plants [32]. This species was introduced to Shanghai in 1935 as an ornamental plant and then escaped into the wild [33] and has since become highly abundant in China [34]. Based on microsatellite and chloroplast locus data, Moran found that *S*. *canadensis* collected from colder locations in Switzerland tended to grow faster at a site of the highest elevation, whereas samples collected from warmer sites did not, indicating the possibility of local adaptation to cold weather [35]. Based on growth chamber and greenhouse experiments, we determined that the phenotypic plasticity of *S*. *canadensis* may have evolved rapidly in regions with different climatic conditions and might have contributed to the spread of this invasive species [22]. However, we also found that individual plasiticty, not local adaptation, plays an important role in the response of *S*. *canadensis* to shade [36]. Recently, *S*. *canadensis* was shown to be tolerant to salinity and to have invaded alkali sandy loams with a significant salt content, such as the Jiuduansha intertidal wetland shoals located at the junction of the Yangtze River and the East China Sea [37] and the polders on the wetland of Hangzhou Bay [38]. With regard to the toxic and selection pressure of salt, we hypothesized that local adaptation might play important role in the invasion success of *S*. *canadensis* into sodic soil. However, there is thus far no experimental evidence. Therefore, in this study, we conducted a greenhouse experiment with replicate cuttings of genets from different populations and addressed three questions: (1) Is the salt tolerance of *S*. *canadensis* populations related to soil salt levels? (2) Do *S*. *canadensis* populations from high salt-level soil grow better than those from low salt-level soil? If not, (3) does the variation in plasticity in response to salt exist within or among populations of *S*. *canadensis*? (4) Is the plasticity of physiological traits correlated with the salt tolerance of *S*. *canadensis*?

## Materials and methods

### Plant species

*Solidago canadensis*, a forb belonging to the Asteraceae family, can produce annual clonal aboveground shoots from persistent belowground rhizomes [34]. This clonal growth leads to dense stands of shoots that reduce native species diversity [34]. The seeds are small, numerous, and wind dispersed, which is necessary for long-distance dispersal [34]. In addition, *S*. *canadensis* is self-incompatible [39].

### Sample collection and propagation of plant materials

In the winter of 2012, rhizome systems were collected from 11 populations of *S*. *canadensis* in China. The populations came from representative habitats; most of the field sites were located near roads and consisted largely of ruderal vegetation. The field sites did not involve any endangered or protected species, and none of the populations were privately owned or under nature protection. No specific permissions were required for these locations. The geographical information and the types of land uses of the populations are shown in Table 1. Soil salinity levels were extracted from the Harmonized World Soil Database at a 0.5-arc-minute spatial resolution from FAO (http://www.fao.org/; detailed information is shown in Table 1). Excess free salts, referred to as soil salinity, is measured as electrical conductivity (EC) or exchangeable sodium percentage (ESP). Among the populations, the salinity levels of 7 were low (EC <4 ds/m or ESP < 6%), whereas those of the other 4 were moderate (EC = 4–8 ds/m or ESP = 6–15%), high (EC = 8–16 ds/m or ESP = 15–25%) or very high (EC > 16 ds/m or ESP > 15%) [40]. Within each population, 3 randomly selected shoot bases with attached rhizomes were dug up and kept moist until replanting. The clonal sprouts of *S*. *canadensis* generate from belowground rhizome fragments; thus, the distances between the shoots were at least 10 m to avoid sampling the same genet twice. All rhizome systems were planted in pots in a common garden at Taizhou University (E 121°17´, N 28°87´) in Linhai City, Zhejiang Province, China. The pots were 30 cm in diameter and 30 cm deep and were filled with a mixed matrix composed of soil, sand and peat soil in a 6:3:1 ratio with a final pH of 6.80±0.10, an organic matter content of 27.66±1.19 g/kg, a total nitrogen content of 361.00±33.00 mg/kg, an available phosphorus content of 8.00±1.14 mg/kg, and an available potassium content of 12.00±1.00 mg/kg. Flow cytometry analysis using a tender leaf showed that individuals from all localities sampled in this study exhibited the same ploidy level (i.e., hexaploidy; 6n = 54).

**Table 1:** Geographical information, types of land use and soil salinity of *Solidago canadensis* populations.

No.	Population abbreviation	Location	Longitude	Latitude	Altitude (m)	Types of land uses	Soil salinity
1	PD	Pudong District, Shanghai City	E121.804°	N31.354°	3	Abandoned farmland	Moderate salinity
2	TZ	Taizhou City, Zhejiang Province	E121.397°	N28.656°	6	Abandoned farmland	Low salinity
3	NT	Nantong City, Jiangsu Province	E120.843°	N32.070°	5	Abandoned farmland	Low salinity
4	WZ	Wenzhou City, Zhejiang Province	E120.607°	N28.126°	4	Abandoned farmland	Low salinity
5	HZ	Xiaoshan District, Hangzhou City, Zhejiang Province	E120.297°	N30.161°	9	Abandoned farmland	Low salinity
6	FZ	Fuzhou City, Fujian Province	E119.359°	N26.098°	19	Abandoned farmland	Low salinity
7	LYG	Lianyungang City, Jiangsu Province	E119.235°	N34.654°	3	Abandoned farmland	Very high salinity
8	WHu	Wuhu City, Anhui Province	E118.387°	N31.342°	16	Garbage dump	Moderate salinity
9	JDZ	Jingdezheng City, Jiangxi Province	E117.166°	N29.318°	40	Green belts	Low salinity
10	JJ	Jiujiang City, Jiangxi Province	E116.283°	N29.985°	18	Abandoned vegetable garden	High salinity
11	WH	Hankou District, Wuhan City, Hubei Province	E114.350°	N30.878°	25	Abandoned farmland	Low salinity

### Salt treatment experiment

The salt treatment experiment was conducted in a greenhouse at Taizhou University under identical light, humidity and temperature conditions in the summer of 2014. The mean daily temperature ranged from 25°C to 34°C. The photosynthetic active radiation is approximately 80% of the strength of natural sunlight. Rosettes with a similar height (mean = 15 cm) were selected and removed from the collected rhizome systems after sprouting and individually planted in pots (16 cm diameter, 14 cm height) containing the mixed matrix. For every population, three genotypes were used as replicates. For every genotype, two rosettes were used for the salt treatment and control. One rosette was planted in each pot. All plant materials used in the different treatments in this study were clonally propagated two years after being commonly planted so that the differences caused by different genotypes and the effect of the mother body were excluded in the experiment. Eight days after planting, 50 ml of 1/8 Hoagland solution was applied to each pot. Ten days after planting, the plant height (H_t1_) and number of leaves (N_t1_) were calculated as the initial data (t1 = 0 day). Plant height was recorded as the distance from the ground to the highest leaf position. To set up the periodic salt treatments, 50 ml of a 300 mmol/L NaCl solution was applied to each pot for two days, followed by 50 ml of tap water for one day, then 50 ml of Hoagland solution for one day. For the control, 50 ml of tap water was applied to each pot for three days, followed by 50 ml of Hoagland solution for one day. The positions of the pots were randomized within the experimental chamber every week to confirm that no position effect occurred.

### Measurements

Twenty-eight days after salt treament, plant height (H_t2_) was measured via ruler with an accuracy of 0.1 cm. The number of leaves (N_t2_) was also recorded. The increase rate in plant height was calculated as (H_t2_-H_t1_)/(t_2_-t_1_) and the rate of increase of the number of leaves was calculated as (N_t2_-N_t1_)/(t_2_-t_1_).

*In situ* photosynthesis measurements were made on the third fully expanded leaf, using a portable photosynthesis-measurement system (LI-6400 XT, Li-COR Inc., Lincoln, NE, USA). Measurements were obtained between 9:00 AM and 11:00 AM under a photosynthetically active radiation of 1,400 μmol m^-2^ s^-1^ (i.e. at light saturation) at a leaf temperature of 25°C, a CO_2_ concentration of 400 ppm, and relative humidity of 70%. Net photosynthetic rate (*P*_n_), stomatal conductance (*g*_s_), transpiration rate (*T*_r_), and intercellular CO_2_ concentration (*C*_i_) were measured.

Three tender leaves per plant were collected and transferred to the lab immediately upon collection. The content of proline was determined via acid ninhydrin colorimetry using L-proline as the standard [41]. The activity of peroxidase was measured by ultraviolet spectrometry [42]. The relative chlorophyll content was measured using a chlorophyll content meter (CCM-200 plus, Opti-Science Inc. Hudson, NH, USA).

Following measurements, plants were harvested and divided into leaves, stems, and roots. Plant material was over-dried (at 105°C for 1 h and then at 80°C until a constant weight was reached). The leaf, stem, and root biomasses were weighed using a balance with a precision of 0.0001 g (Shanghai Jingtian Electronic Instrument Co., Ltd, Shanghai, China). Total biomass and root/shoot ratios were calculated.

### Statistical analyses

The tolerance index (TI) in terms of the root, shoot, total biomass and plant height was calculated for each population, i.e., the index of the plants under salinity stress divided by the index of the plants in the control [43]. The phenotypic plasticity index was calculated based on the difference between the maximum mean and minimum mean divided by the maximum mean [44].

All data are shown as the mean ± standard error. A t-test was applied to test the effect of the salt treatment on the plants. A two-way analysis of variance (ANOVA) was applied to test the effects of salt, population and their interaction on physiological traits, with the treatment as a fixed factor and the population and genotype (nested to population) as random factors. One-way ANOVA was applied to test the differences in plasticity among different populations. A variance component analysis was conducted to calculate the differentiation coefficient of the phenotypic plasticity index within or between different populations. The relationships between phenotypic plasticity and the salt tolerance index were evaluated separately by Pearson’s correlation analysis. The relationships between the salt tolerance index and soil salinity level were evaluated using Spearman’s rho value through a nonparametric test (binomial test). All statistical analyses were conducted using SPSS 16.0 software.

## Results

### Effects of salinity on growth and salt tolerance

**Fig 1:**
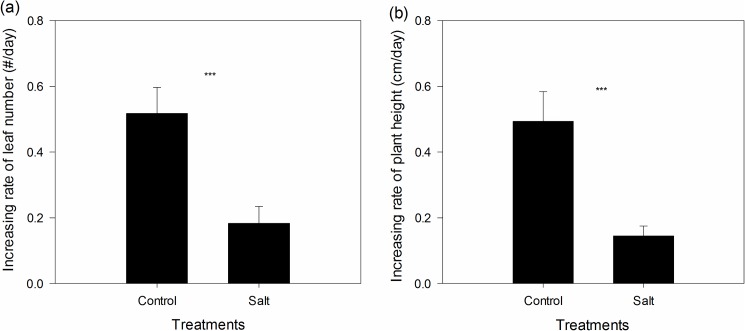
Effect of salt treatment on the rate of increase in the number of leaves (a) and plant height (b). Values are shown in mean ± standard error (SE). ***, indicates that the difference between the control and the salt treatment is significant at *p*<0.001.

**Fig 2:**
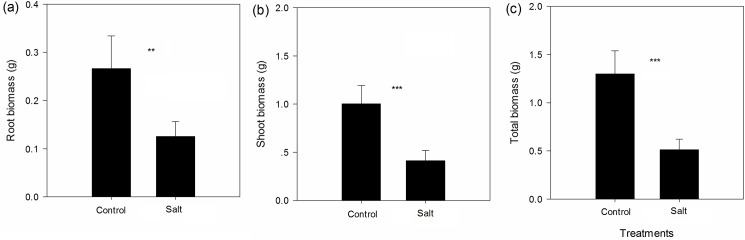
Effect of salt treatment on root biomass (a) shoot biomass (b) and total biomass (c). Values are shown in mean ± standard error (SE). *, **, ***, indicate that the difference between the control and salt treatment is significant at *p*<0.05, *p*<0.01 and *p*<0.001, respectively.

**Fig 3:**
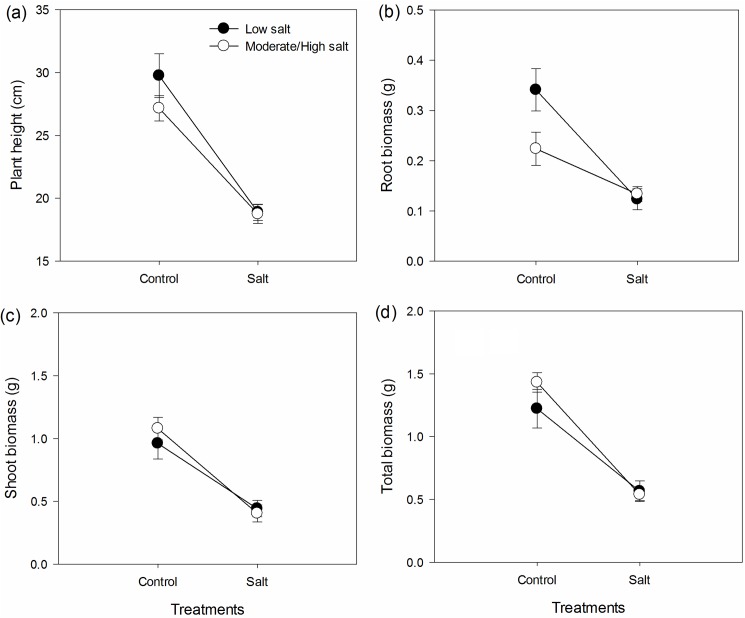
Effect of salt treatment on the plant height (a) and root (b), shoot (c) and total biomass (d) of *S*. *canadensis* populations from moderate/severe salt-level soil and low salt-level soil. Values are shown in mean ± standard error (SE).

The salt treatment significantly inhibited the rate of increase in the number of leaves (Fig 1A, paired *t* value = 8.987, *p*<0.001) and the rate of increase in plant height (Fig 1B, paired *t* value = 7.245, *p*<0.001). Salt treatment also significantly reduced the root biomass (Fig 2A, paired *t* value = 4.306 *p* = 0.002), shoot biomass (Fig 2B, paired *t* value = 6.597, *p*<0.001) and total biomass (Fig 2C, paired *t* value = 6.733, *p*<0.001). Under salt treatment, the root, shoot, and total biomass and plant height of *S*. *canadensis* populations from moderate/severe salt-level soil were similar to those of *S*. *canadensis* populations from low salt-level soil (Fig 3).

Based on the root, shoot and total biomass, the salt tolerance of *S*. *canadensis* of the NT population was highest, whereas the salt tolerance of the LYG population was lowest. Based on plant height, the salt tolerance of the HZ and TZ populations was highest and lowest, respectively (Table 2). However, no significant differences were found among the populations (*p*>0.05). Nonparametric correlations analysis showed no significant correlation between salt tolerance indices and soil salinity levels (Table 2).

**Table 2:** Mean ± standard error (SE) of the salt tolerance for each trait of *Solidago canadensis* and nonparametric correlations with soil salinity levels.

Population	Plant height	Shoot biomass	Root biomass	Total biomass
PD	0.71±0.14	0.33±0.03	0.59±0.20	0.38±0.17
TZ	0.43±0.03	0.43±0.21	0.50±0.29	0.42±0.26
NT	0.85±0.10	0.94±0.06	0.77±0.09	0.88±0.08
WZ	0.50±0.07	0.36±0.08	0.43±0.06	0.34±0.04
HZ	0.90±0.18	0.35±0.14	0.39±0.08	0.36±0.12
FZ	0.42±0.01	0.47±0.03	0.62±0.25	0.49±0.08
LYG	0.54±0.03	0.14±0.03	0.24±0.07	0.18±0.07
WHu	0.62±0.03	0.52±0.18	0.47±0.17	0.62±0.31
JDZ	0.58±0.10	0.39±0.21	0.35±0.16	0.39±0.0.21
JJ	0.72±0.15	0.50±0.27	0.52±0.30	0.50±0.28
WH	0.57±0.13	0.36±0.14	0.47±0.19	0.37±0.15
Spearman’s rho (*p* value)	0.171 (0.425)	-0.329 (0.116)	-0.207 (0.331)	-0.286 (0.175)

### Effects of salinity on physiological traits, root/shoot ratio and their plasticities

The salt treatment significantly increased peroxidase activity, proline content and root/shoot ratio, and significantly decreased the net photosynthetic rate, stomatal conductance, and transpiration rate; however, the salt treatment had no significant effect on the intercellular CO_2_ concentration (Table 3). Two-way ANOVA showed that the salt treatment had a significant effect on the root/shoot ratio and the physiological traits of *S*. *canadensis*, except for the intercellular CO_2_ concentration; In contrast, the population, genotype and the interaction salt × population had no significant effect on all traits. The proline content showed the strongest plasticity, whereas the intercellular CO_2_ concentration and root/shoot ratio showed the weakest plasticity (Table 3). Most of the variation in plasticity existed within populations and not among populations, except for the root/shoot ratio (Table 3).

**Table 3:** Mean ± standard error (SE) of each physiological trait under the control and salt treatment and the mean ± SD and differentiation coefficient (*V*st) of the phenotypic plasticity index (PPI) for each physiological trait of *Solidago canadensis*. Different small letters on the same line indicate that the difference between the control and salt treatment was significant. The results of two-way ANOVA and the population differentiation coefficient are listed.

	Effect of salinity						Phenotypic plasticity	
Trait	Control (mean±SE)	Salt (mean±SE)	*F*_salt_	*F*_pop_	*F*_salt×pop_	*F*_geno_	PPI (mean±SD)	*V*st
Proline content	18.10±15.81a	342.45±174.94b	80.61***	1.30	1.05	0.69	0.93±0.07	0.44
Peroxidase activity	2.10±0.81a	3.13±1.94b	7.78*	0.82	1.34	1.36	0.37±0.23	0.48
Net photosynthetic rate	11.35±1.96a	6.21±2.57b	64.16***	1.83	1.09	0.71	0.47±0.21	0.30
Stomatal conductance	0.70±0.17a	0.31±0.16b	89.89***	0.60	0.60	0.29	0.56±0.22	0.28
Intercellular CO_2_ concentration	344.66±7.87a	341.42±13.79a	2.53	10.11	0.58	0.53	0.03±0.03	0.56
Transpiration rate	7.68±1.41a	4.97±2.01b	36.93***	11.10	0.78	0.33	0.37±0.27	0.39
Chlorophyll content	1.04±0.22a	0.57±0.18b	71.26***	5.57	1.31	0.34	0.46±0.19	0.48
Root/shoot ratio	0.28±0.05b	0.34±0.06a	3.75*	0.99	1.01	0.78	0.28±0.03	0.97

Note:

*, *** indicate significance at the 0.05 and 0.001 levels, respectively.

### Correlation between the salt tolerance index and the plasticity of physiological traits and the root/shoot ratio

Pearson’s correlation analysis revealed significant correlations between the plasticity of proline content and the salt tolerance index based on plant height; the plasticity of the intercellular CO_2_ concentration and salt tolerance indices based on total biomass, shoot biomass and plant height; and the plasticity of the chlorophyll content and the salt tolerance index based on plant height (Table 4).

**Table 4:** Pearson’s correlation between the plasticity index of physiological traits and the salt tolerance index. The *p* values are listed in parentheses. The figures in bold indicate significant correlations.

Salt tolerance index	Proline content	Peroxidase activity	Net photosynthetic rate	Stomatal conductance	Intercellular CO_2_ concentration	Transpiration rate	Chlorophyll content	Root/shoot ratio
Total biomass	-0.003(0.987)	-0.125(0.550)	0.071(0.737)	0.231(0.266)	**0.417(0.038)**	0.196(0.347)	-0.245(0.248)	-0.074(0.724)
Root biomass	-0.099(0.639)	-0.032(0.880)	0.261(0.208)	0.325(0.113)	0.166(0.427)	0.214(0.304)	-0.192(0.369)	0.065(0.758)
Shoot biomass	-0.020(0.926)	-0.119(0.571)	0.027(0.899)	0.197(0.346)	**0.414(0.039)**	0.151(0.471)	-0.242(0.254)	-0.014(0.947)
Plant height	**-0.570(0.003)**	0.163(0.435)	-0.215(0.302)	-0.103(0.625)	**0.500(0.011)**	-0.135(0.519)	**-0.431(0.036)**	-0.060(0.775)

## Discussion

Salinity is a limiting environmental factor that impairs plant growth and development [45]. The toxicity of salt results in strong selection pressure on the salt tolerance of plants and different ecotypes, and genetic mutants with salt tolerance have evolved during the adaption to salt-affected soil [46]. In our greenhouse experiment, we did not find that *S*. *canadensis* from higher saline-level soil performed better than plants from low saline-level soil; furthermore, the salt tolerance indices in terms of root, shoot, total biomass and height did not show any significant correlations with the soil salinity levels. Our results suggest the absence of local adaption in the existing populations of *S*. *canadensis* collected from soils with medium, high and extremely high salinity, which was contrary to our original hypothesis. Similar phenomena have been observed in the perennial clonal plant *Leymus chinensis* in response to water [7] and the invasive plant *Fallopia japonica* in response to salt [3], but not in invasive *Ipomoea cairica* in response to salt [47] and invasive *S*. *canadensis* in response to cold [35]. Richards et al. hypothesized that local adaptation would occur in *F*. *japonica* established in salt marsh habitats because of increased salt tolerance compared to those established in the more common roadside habitat. However, these authors found that *F*. *japonica* from the salt marsh habitats did not perform better in the salt treatment, suggesting that selection by the salt content of the marsh habitat did not generate genotypes adapted to high salinity [3]. Having conducted a meta-analysis of local adaptation in plants, Leimu and Fischer suggested that local adaption is less common in plant populations than is generally assumed [48]. The occurrence and strength of local adaptation by plants in response to environmental variation may be dependent on species as well as on the life history, population size, and study characteristics [49].

The absence of local adaptation of *S*. *canadensis* in sodic soils might be due to three reasons. The first is the extremely high plasticity of *S*. *canadensis*, which might cover the effect of genotype variation. The second is the relatively short invasion history in colonization of sodic soil. Although differences in soil salinity levels existed in the different population, the short invasion history and weak selection pressure might not have contributed to the local adaptation of *S*. *canadensis* populations. The third is the population origin and non-quantitative measurment of soil salinity, which might also be the main limitation of the experiment design. The populations used in the study were collected from normal habitats and, not from coastal areas, where the difference in soil salinity levels might be greater. In addition, we extracted the soil salinity level data from the Harmonized World Soil Database at a 0.5-arc-minute spatial resolution from FAO, which might not be consistent with the local habitat conditions from which the *S*. *canadensis* individuals were collected. The relatively small difference in soil salinity levels and the relatively coarse broad-scale information of soil salinity levels might have affected the corrlation between salt tolerance indices and soil salinity levels. The existence of local adaption to cold temperature has been reported [35]. Thus, although no clear evidence for local adaptation was found in this study, we should still be careful in drawing a general conclusion about the absence of local adaption of *S*. *canadensis* in colonization of sodic soils. Further study should involve collection of plants from sodic soils with clear and relatively greater differences in salinity levels, direct measurement of the soil salinity content and calculation of the relationship between salt tolerance indices and the quantitative sat content.

Plasticity for ecologically important traits may enhance the ability to withstand adverse environmental conditions or to respond positively to favorable conditions, thus promoting invasiveness [49]. It has been well documented that phenotypic plasticity may be a common trait of plant invaders (reviewed by Richards et al. [19]. Richards et al. demonstrated that plasticity in salt tolerance traits might allow invasive *Fallopia japonica* and *F*. *× bohemica* to live in saline habitats without any specific adaptation to tolerate salt [3]. Our previous study showed that the phenotypic plasticity of *S*. *canadensis* is high in response to temperature and water availability [22]. In the present study, we observed strong plasticity of the proline content, intermediate plasticity of the net photosynthetic rate, stomatal conductance and relative chlorophyll content, and relatively weaker plasticity of the transpiration rate and the activity of peroxidase. Pearson’s correlation analysis revealed a significantly negative correlation between the plasticity of proline content or chlorophyll content and the salt tolerance index based on plant height and a significant positive correlation between the plasticity of the intercellular CO_2_ concentration and salt tolerance indices based on total biomass, shoot biomass and plant height. Our experimental evidence suggests that phenotypic plasticity may be the primary adaptive strategy for invasive *S*. *canadensis* with regard to salinity tolerance. These results show that the ability of *S*. *canadensis* to maintain higher chlorophyll content, lower proline content and higher intercellular CO_2_ concentration enables greater growth at higher levels of salinity.

Although the variation of phenotypic plasticity among invading populations has rarely been documented [50], some studies have indicated that variation of plasticity among introduced populations may allow an invader to evolve greater plasticity, resulting in the colonization of more diverse habitats and plant communities [17, 49, 50]. A previous study showed that individual plasticity, not local adaptation, played a more prominent role in the shade response of invasive *S*. *canadensis* populations under similar light conditions [36]. In the present study, we found that most of the variation in the plasticity of the physiological traits of *S*. *canadensis* occurred among individuals within populations and not among populations, indicating that the potential of *S*. *canadensis* to evolve greater tolerance or adaptation to saline soil was small. Similar results were obtained for the clonal perennial plant *Leymus chinensis* [7] but not the invasive plant *Microstegium vimineum* [49] and *Ipomoea cairica* [47]. In addition, we also found taht most of the variation in the plasticity of the root/shoot ratio of *S*. *canadensis* occurred among populations, indicating that the evolutionary potential of the variation in root/shoot ratio was large. However, we did not find a significant correlation between salt tolerance indices and the plasticity of the root/shoot ratio, which might also be due to the short colonization history of *S*. *canadensis* in saline habitats or the small difference in soil salinity levels.

Taken together, these findings indicate that individual plasticity might be more important than local adaptation in affecting physiological traits and salt tolerance across the distribution of the species. We predict that *S*. *canadensis*, which is dominant on broadsides [34], might have the potential to become a serious problem in sodic soils, including coastal areas, in the future. More attention should focus on monitoring the occurrence of *S*. *canadensis* on sodic soils, and timely prevention should be implemented to control new invaders.
